# Compreendendo as Alterações Cardíacas na Lipodistrofia Parcial Familiar: Insights da Ecocardiografia

**DOI:** 10.36660/abc.20240305

**Published:** 2024-07-01

**Authors:** Silvio Henrique Barberato

**Affiliations:** 1 CardioEco Centro de Diagnóstico Cardiovascular Curitiba PR Brasil CardioEco Centro de Diagnóstico Cardiovascular, Curitiba, PR – Brasil; 2 Quanta Diagnóstico – Ecocardiografia Curitiba PR Brasil Quanta Diagnóstico – Ecocardiografia, Curitiba, PR – Brasil

**Keywords:** Ecocardiografia, Lipodistrofia, Testes de Função Cardíaca

As lipodistrofias são um grupo diversificado de doenças caracterizada por perda de tecido adiposo no corpo. Consequentemente, os lipídios são armazenados em células não adiposas, levando a importantes consequências metabólicas, como esteatose hepática e resistência à insulina, e aumento do risco de doenças cardiovasculares devido à aterosclerose prematura.^1^ As lipodistrofias podem ser classificadas em congênitas ou adquiridas e generalizadas ou parciais, dependendo da extensão do distúrbio na distribuição de gordura.^1^ Ao contrário da lipodistrofia congênita generalizada (LCG), os pacientes com lipodistrofia parcial familiar (LP) não apresentam uma falta generalizada de gordura por todo o corpo, mas sim uma perda localizada de gordura nos membros e no tronco. A LP é uma doença genética rara, frequentemente acompanhada por mutações no gene da lamina A/C (LMNA), pertencente ao complexo grupo das laminopatias que podem levar a distrofias musculares e cardíacas, neuropatias e síndromes de envelhecimento precoce.^2,3^ Devido à raridade das lipodistrofias e a gama fenotípica de manifestações cardíacas, apenas estão disponíveis relatos de casos e estudos com número limitado de pacientes, em sua maioria restritos a indivíduos com LCG.

A cardiomiopatia associada à LP familiar permanece pouco explorada, apesar do seu potencial de causar mortalidade precoce. Embora a aterosclerose prematura^4^ e as arritmias cardíacas sejam comuns,^5^ o impacto direto das mutações genéticas na estrutura e função cardíaca não é claro. Relatos anteriores descreveram diferentes fenótipos de cardiomiopatias neste grupo de pacientes, variando de hipertrofia isolada do ventrículo esquerdo (VE) e cardiomiopatia hipertrófica a cardiomiopatia dilatada.^4^

A ecocardiografia é uma ferramenta clínica não invasiva e amplamente disponível que desempenha papel fundamental na caracterização de alterações morfológicas e funcionais cardíacas.^6^ O estudo de Romano et al.,^7^ publicado nesta edição da ABC Cardiol, elucida alterações ecocardiográficas em pacientes assintomáticos com LP familiar e fornece insights sobre alterações cardíacas subclínicas que podem ocorrer nesta doença rara. O estudo é bem estruturado, destaca a lacuna de conhecimento sobre alterações cardíacas na LP familiar e contribui para a compreensão do fenótipo cardíaco e suas possíveis implicações clínicas neste grupo de indivíduos.

Com o objetivo de caracterizar a morfologia e a função cardíaca em pacientes com LP familiar sem sintomas cardíacos por meio da ecocardiografia, encontraram-se diferenças significativas entre pacientes com LP e controles. Os pacientes LP apresentaram maior massa do VE e tamanho do átrio esquerdo, bem como menores índices de função diastólica, independentemente dos níveis de pressão arterial sistólica. Embora a fração de ejeção do VE tenha sido normal em ambos os grupos, uma proporção considerável de pacientes com LP (75%, ou nove em cada doze) apresentou deformação reduzida na análise de *strain* por *speckle-tracking*, o que indica disfunção subclínica precoce do VE. Embora o *strain* longitudinal global médio (SGL) dos pacientes com LP não tenha sido estatisticamente diferente dos controles, o subgrupo de pacientes com deformação reduzida apresentou alterações mais profundas na geometria cardíaca e na função diastólica. Como destacam os autores, esse achado lembra outras cardiomiopatias metabólicas e pode eventualmente evoluir para insuficiência cardíaca com fração de ejeção preservada. É digno de nota que quase 80% dos pacientes do estudo foram submetidos a um teste genético, que revelou que todos eram portadores de variantes no gene LMNA. É claro que o estudo tem algumas limitações, principalmente devido ao seu desenho e à raridade da doença. O pequeno tamanho da amostra e a natureza transversal da investigação restringem a exploração de consequências a longo prazo e informações prognósticas. Embora diversas diferenças foram encontradas entre as variáveis ecocardiográficas dos grupos LP e controle, houve considerável sobreposição entre elas. Além disso, as medidas do SLG foram obtidas apenas para 21 dos 29 indivíduos LP.^7^

Os achados de Romano et al.^7^ levantam a questão de saber se a detecção precoce de alterações ecocardiográficas em pacientes com LP, mesmo na ausência de sintomas, poderia eventualmente permitir uma intervenção oportuna e prevenir resultados adversos. O estudo agrega conhecimento valioso sobre o envolvimento cardíaco na LP familiar, contribuindo para a compreensão do espectro da doença e ressaltando a importância da avaliação cardíaca de rotina no manejo da LP. Estudos prospectivos com coortes maiores são necessários para elucidar a evolução clínica e os fatores de risco para eventos cardíacos adversos em pacientes com LP.
