# Supplementary material for: Compreendendo as Alterações Cardíacas na Lipodistrofia Parcial Familiar: Insights da Ecocardiografia
Source: Arq Bras Cardiol. 2024 Jul 1;121(6):e20240305. [Article in Portuguese] doi: 10.36660/abc.20240305 (PMC12080607; doi:10.36660/abc.20240305)
Supplement: Translation

# Translation: Understanding Cardiac Alterations in Familial Partial Lipodystrophy: Insights from Echocardiography

**DOI:** 10.36660/abc.20240305i

**Authors:** Silvio Henrique Barberato

**Affiliations:** 1 Curitiba PR Brazil CardioEco Centro de Diagnóstico Cardiovascular, Curitiba, PR – Brazil; 2 Curitiba PR Brazil Quanta Diagnóstico – Ecocardiografia, Curitiba, PR – Brazil

**Keywords:** Lipodystrophy, Heart Function Tests, Echocardiography

Lipodystrophies are a diverse group of diseases characterized by a loss of fatty tissue in the body. Consequently, lipids are stored in non-fat cells, leading to major metabolic consequences, such as liver steatosis and insulin resistance, and increased risk of cardiovascular diseases due to premature atherosclerosis.^1^ Lipodystrophies can be classified as congenital or acquired, and generalized or partial, depending on the extent of fat distribution disturbance.^1^ Unlike congenital generalized lipodystrophy (CGL), patients with familial partial lipodystrophy (PL) do not have a widespread lack of fat throughout the body but rather a localized loss of fat in limbs and trunk. PL is a rare genetic disorder often accompanied by mutations in the lamin A/C gene (LMNA), belonging to the complex group of laminopathies that can lead to muscular and cardiac dystrophies, neuropathies, and syndromes of premature aging.^2,3^ Due to the rarity of the lipodystrophies and the phenotypic range of cardiac manifestations, only case reports and studies with limited numbers of patients, mostly restricted to individuals with CGL, are available.

Cardiomyopathy associated with familial PL remains underexplored despite its potential to cause early mortality. While premature atherosclerosis^4^ and cardiac arrhythmias are common,^5^ the direct impact of gene mutations on cardiac structure and function is unclear. Previous reports have described different phenotypes of cardiomyopathies in this group of patients, ranging from isolated left ventricular (LV) hypertrophy and hypertrophic cardiomyopathy to dilated cardiomyopathy.^4^

Echocardiography is a widely available, non-invasive clinical tool that plays a fundamental role in characterizing cardiac morphologic and functional alterations.^6^ The study by Romano et al.^7^ published in this issue of ABC Cardiol, sheds light on echocardiographic abnormalities in asymptomatic patients with familial PL and provides insights into subclinical cardiac changes that can occur in this rare disease. The study is well-structured, highlights the knowledge gap regarding cardiac alterations in familial PL, and adds to the understanding of the cardiac phenotype and its conceivable clinical implications in this group of individuals.

Aiming to characterize cardiac morphology and function in familial PL patients without cardiac symptoms using echocardiography, they found significant differences between PL patients and controls. PL patients exhibited higher LV mass and left atrium size, as well as lower indices of diastolic function, independent of systolic blood pressure levels. Although LV ejection fraction was normal in both groups, a considerable proportion of PL patients (75%, or nine out of twelve) showed reduced deformation on speckle-tracking strain analysis, which indicates early LV subclinical dysfunction. Although the average global longitudinal strain (GLS) of PL patients was not statistically different from the controls, the subgroup of patients with reduced deformation showed more profound changes in cardiac geometry and diastolic function. As the authors highlight, this finding is reminiscent of other metabolic cardiomyopathies and may eventually progress to heart failure with preserved ejection fraction. Of note, almost 80% of the study patients underwent a genetic test, which revealed that all of them carried variants in the LMNA gene. Of course, the study has some limitations, mostly due to its design and the rarity of the disease. The small sample size and the cross-sectional nature of the investigation restrict the exploration of long-term consequences and prognostic information. Even though several differences were found between the echocardiographic variables of the PL and control groups, there was considerable overlap between them. Moreover, GLS measurements were only obtained for 21 out of the 29 PL individuals.^7^

The findings of Romano et al.^7^ raise the question of whether the early detection of echocardiographic alterations in PL patients, even in the absence of symptoms, could eventually enable timely intervention and prevent adverse outcomes. The study adds valuable knowledge about cardiac involvement in familial PL, contributing to the understanding of the disease spectrum and underscoring the importance of routine cardiac assessment in PL management. Prospective studies with larger cohorts are necessary to elucidate the clinical course and risk factors for adverse cardiac events in PL patients.
